# Clinical meaning of serum trimethylamine oxide, N-terminal-pro-brain natriuretic peptide, hypoxia-inducible factor-1a and left ventricular function and pregnancy outcome in patients with pregnancy-induced hypertension

**DOI:** 10.5937/jomb0-37030

**Published:** 2023-03-15

**Authors:** Ying Wu, Yue Wu, Lihong Duan, Chunhui Xiao, Zeya Ren, Yuntai Liang

**Affiliations:** 1 Shijiazhuang Fourth Hospital, Department of Obstetrics, Hebei Province, Shijiazhuang City, China; 2 Hebei Provincial Key Laboratory of Maternal Fetal Medicine, Hebei Province, China; 3 Chinese People's Liberation Army 66172 Army Hospital, Department of Internal Medicine, Shijiazhuang City, Hebei Province, China

**Keywords:** gestational hypertension, trimethylamine oxide, N-terminal-pro-brain natriuretic peptide, hypoxia-inducible factor-1a, left ventricular function, pregnancy outcome, gestacijska hipertenzija, trimetilamin oksid, N-terminalni-pro-mozak natriuretski peptid, faktor koji izaziva hipoksiju-1a, funkcija leve komore, ishod trudnoće

## Abstract

**Background:**

To figure out the clinical meaning of serum trimethylamine oxide (TMAO), N-terminal-pro-brain natriuretic peptide (NT-proBNP) and hypoxia-inducible factor-1a (HIF-1a) with left ventricular function and pregnancy outcome in patients with pregnancy-induced hypertension.

**Methods:**

From January 2018 to October 2020, 117 patients with gestational hypertension were taken as the research objects and grouped into the gestational hypertension (pregnancy-induced hypertension, 55 cases), mild preeclampsia (mild PE, 43 cases) and severe preeclampsia (severe PE, 19 cases) in the light of the severity of the disease. Analysis of the relation of serum TMAO, NT-proBNP and HIF-1a with the severity of disease and cardiac function indexes in patients with gestational hypertension was conducted. All patients were followed up to the end of pregnancy, and the predictive value of serum TMAO, NT-proBNP and HIF-1a on pregnancy outcome in patients was analyzed.

**Results:**

Serum TMAO and NT-proBNP of patients were elevated, while HIF-1a was reduced with the severity of the disease (P < 0.05). Serum TMAO and NT-proBNP in patients with gestational hypertension were positively correlated but HIF-1a was negatively correlated with the severity of the disease (P < 0.05). Left ventricular end-diastolic volume (LVEDV) and left ventricular end-systolic volume (LVESV) were elevated in gestational hypertension patients, while ejection fraction (LVEF) was reduced with the severity of disease (P < 0.05). Serum TMAO, NT-proBNP and HIF1a were associated with LVEDV, LVESV and LVEF values in patients with gestational hypertension (P < 0.05). Serum TMAO and NT-proBNP were elevated but HIF-1a was reduced in patients with a poor pregnancy outcome (P < 0.05). The AUC of the combined detection of serum TMAO, NT-proBNP and HIF-1a on pregnancy outcome was greater (P < 0.05).

**Conclusions:**

Serum TMAO, NT-proBNP and HIF-1a in patients with gestational hypertension are associated with disease severity and cardiac function, and have predictive and evaluative values for disease severity and pregnancy outcome.

## Abstract (sr)

**Uvod:**

Cilj je bio da se otkrije kliničko značenje serumskog trimetilamin oksida (TMAO), N-terminalnog pro-moždanog natriuretskog peptida (NT-proBNP) i faktora indukovanog hipoksijom-1a (HIF-1a) sa funkcijom leve komore i ishodom trudnoće u pacijenti sa hipertenzijom izazvanom trudnoćom.

**Metode:**

Od januara 2018. do oktobra 2020. godine, 117 pacijentkinja sa gestacijskom hipertenzijom uzeto je kao predmet istraživanja i grupisano u gestacijsku hipertenziju (hipertenzija izazvana trudnoćom, 55 slučajeva), blagu preeklampsiju (blaga PE, 43 slučaja) i tešku preeklampsiju (teška PE, 19 slučajeva) u svetlu težine bolesti. Urađena je analiza odnosa serumskih TMAO, NT-proBNP i HIF-1a sa težinom bolesti i indeksima srčane funkcije kod pacijenata sa gestacionom hipertenzijom. Sve pacijentkinje su praćene do kraja trudnoće i analizirana je prediktivna vrednost serumskih TMAO, NT-proBNP i HIF-1a na ishod trudnoće kod pacijenata.

**Rezultati:**

TMAO i NT-proBNP u serumu pacijenata su bili povišeni, dok je HIF-1a smanjen sa težinom bolesti (P < 0,05). TMAO i NT-proBNP u serumu kod pacijenata sa gestacionom hipertenzijom bili su u pozitivnoj korelaciji, ali je HIF-1a bio u negativnoj korelaciji sa težinom bolesti (P < 0,05). Krajnji dijastolni volumen leve komore (LVEDV) i end-sistolni volumen leve komore (LVESV) bili su povišeni kod pacijenata sa gestacionom hipertenzijom, dok je ejekciona frakcija (LVEF) smanjena sa težinom bolesti (P < 0,05). TMAO, NT-proBNP i HIF-1a u serumu su bili povezani sa vrednostima LVEDV, LVESV i LVEF kod pacijenata sa gestacionom hipertenzijom (P < 0,05). TMAO i NT-proBNP u serumu su bili povišeni, ali je HIF-1a smanjen kod pacijenata sa lošim ishodom trudnoće (P < 0,05). AUC kombinovane detekcije TMAO, NT-proBNP i HIF-1a u serumu na ishod trudnoće bila je veća (P < 0,05).

**Zaključak:**

Serumski TMAO, NT-proBNP i HIF-1a kod pacijenata sa gestacionom hipertenzijom povezani su sa težinom bolesti i srčanom funkcijom i imaju prediktivne i evaluativne vrednosti za težinu bolesti i ishod trudnoće.

## Introduction

Gestational hypertension causes proteinuria, edema, hypertension and even eclampsia in pregnant women during pregnancy. It often takes place after 20 weeks of pregnancy. In severe cases, it can endanger the health of mothers and babies, and even result in death. Systemic arteriolar spasm in patients with gestational hypertension results in hypoxic metabolism in systemic tissues, and the body is in a hypoxic environment [1]. Hypoxia-inducible factor-1α (HIF-1α) is a regulator of cells and tissues adapting to a hypoxic environment. It can take part in the formation of inflammatory response, and take on a momentous function in the formation of endothelial factor and the proliferation of smooth muscle cells in a hypoxic environment [2] [3]. Trimethylamine-N-oxide (TMAO) is a metabolite of intestinal flora, and its abnormality can motivate vascular inflammation and oxidative stress. TMAO is a latent risk for atherosclerosis and cardiometabolic diseases [4]. N-terminal pro brain natriuretic peptide (NT-proBNP) is a crucial serum factor in the vascular system. It is an N-terminal precursor fragment after the cleavage of brain natriuretic peptide premonomer. When the vascular wall pressure is overloaded, it can motivate the synthesis and release of BNP precursor [5]. It is clinically believed that NT-proBNP is closely linked with the progression of cardiovascular disease. However, no report clearly points out its association with serum TMAO and HIF-1α and cardiac function and pregnancy outcome in patients with gestational hypertension. Therefore, this research aims to figure out the clinical meaning of serum TMAO, NT-proBNP, HIF-1α, left ventricular function and pregnancy outcome in patients with pregnancy-induced hypertension, offering a reference for the clinical evaluation of the disease.

## Materials and methods

### Clinical data

From January 2018 to October 2020, 117 patients with gestational hypertension were selected as the research objects and grouped into the gestational hypertension (the PIH, 55 cases), mild preeclampsia (the mild PE, 43 cases), and severe preeclampsia (the severe PE, 19 cases) in the light of the severity of gestational hypertension according to the seventh edition of *Obstetrics and Gynecology* [6]. Inclusion criteria: Meeting the diagnostic criteria for gestational hypertension in the *Guidelines for the Diagnosis and Treatment of Hypertensive Diseases in Pregnancy* (2015) [7]; Patients with complete clinical data; Patients with singleton pregnancy. Exclusion criteria: Patients combined with abnormal liver and kidney function; Prone to hypertension before pregnancy; combined obstetric complications; combined gestational diabetes mellitus; combined inflammatory diseases; multiple pregnancy. No clear difference exhibited in general data among the three groups (*P *> 0.05, Table 1).

**Table 1:** Comparison of general data of patients with different severity

Groups	the PIH<br>(n=55)	the mild PE<br>(n=43)	the severe PE<br>(n=19)	χ^2^/F	*P*
Age (years)	31.62±3.10	32.08±3.29	31.19±3.37	0.569	>0.05
Gestational Week (Weeks)	33.19±2.57	32.74±2.85	32.23±3.01	0.961	>0.05
Pregnancy (times)	1.82±0.23	1.75±0.21	1.72±0.22	2.041	>0.05
Parity (times)	1.27±0.25	1.33±0.22	1.42±0.27	2.875	>0.05
Body mass index (kg/m^2^)	23.19±2.75	23.58±2.89	24.01±2.94	0.663	>0.05
Total cholesterol (mmol/L)	4.71±0.78	4.92±0.85	4.69±0.76	1.005	>0.05
Triglycerides (mmol/L)	1.38±0.21	1.45±0.19	1.32±0.24	2.955	>0.05
History of drinking (cases)	5	3	2	0.251	>0.05
Smoking history (cases)	1	1	1	0.692	>0.05
Family history of hypertension (cases)	3	2	1	0.031	>0.05
Education (cases)					
Senior high school and above	34	24	11	0.373	>0.05
Junior high school and below	21	19	8		

### Methods

Detection of serum TMAO, NT-proBNP and HIF-1α: 3 mL of venous blood was collected when patients woke up in the morning within 24 h of enrollment and serum was separated. Mitsubishi PATH-FAST MITSUBISHI chemical spectrophotometer was applied for detecting serum NT-proBNP with chemiluminescence immunoassay in patients. ELISA method was utilized to test TMAO and HIF-1α in patients, and the kit was offered by Shanghai Jinma Experimental Equipment Co., Ltd.

Detection of cardiac function indicators: Siemens SC2000 color ultrasound diagnostic apparatus (4V1c/4Z1c probe, frequency of 1-4MHz) was used. Patients were conventionally connected to lead ECG, and conventional two-dimensional echocardiography and RT-3DE examination were performed in appropriate posture. The patient was in the left lateral decubitus position, the 4Z1c probe and the apical four-chamber view were selected, and the display effect was adjusted to clearly show the left ventricular wall. The full-volume three-dimensional images of three consecutive cardiac cycles were collected and processed by SC2000WP workstation. The in-machine analysis software eSie LVA was applied to measure left ventricular end-diastolic volume (LVEDV), left ventricular end systolic volume (LVESV) and left ventricular ejection fraction (LVEF).

Pregnancy outcome grouping: All patients were followed up to the end of pregnancy and divided into a better pregnancy outcome group and a worse pregnancy outcome group according to the pregnancy outcome. Adverse pregnancy outcomes mainly included stillbirth, miscarriage, and preterm birth.

### Observation indicators

The serum TMAO, NT-proBNP and HIF-1α of PIH, mild PE and severe PE were compared, and their correlation with disease severity and evaluation value of disease severity were analyzed. The serum TMAO, NT-proBNP and HIF-1α in patients with better pregnancy outcome and poor pregnancy outcome were compared, and the predictive value of combined detection on pregnancy outcome was analyzed.

### Statistical processing

SPSS22.0 software was applied for processing data, and enumeration data were shown in %, and compared by χ^2^ test; Measurement data were illustrated by (x̅ ± s) after normality test, and comparison of differences between two groups was done by t test. One-way analysis of variance was applied for comparison of the differences of multiple groups. Spearman test was applied to analyze the correlation between serum TMAO, NT-proBNP, HIF-1α and the severity of gestational hypertension. ROC curve analysis was employed for combined detection of estimates of the severity of gestational hypertension. *P *< 0.05 emphasized obvious statistical meaning.

## Results

### Comparison of serum TMAO, NT-proBNP and HIF-1α in patients with different severity

**Figure 1:**
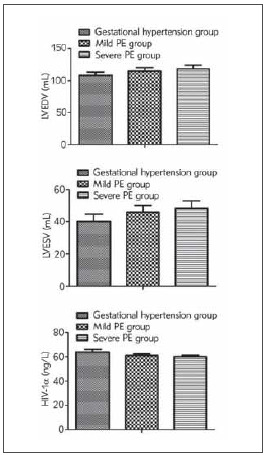
Comparison of serum TMAO, NT-proBNP and HIF-1α in patients with different severity. Comparison of TMAO, NT-proBNP and HIF-1α among the three groups, F=56.834, 2456.007, 93.805, *P* < 0.05

Serum TMAO and NT-proBNP were positively associated with disease severity in patients with gestational hypertension, but HIF-1α was negatively correlated with disease severity (*P* < 0.05, Figure 1).

### Correlation analysis of serum TMAO, NT-proBNP, HIF-1α and severity of gestational hypertension

**Figure 2:**
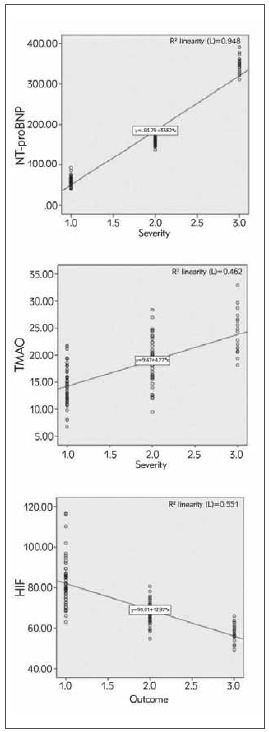
Correlation analysis of serum TMAO, NT-proBNP, HIF-1α and severity of gestational hypertension

Serum TMAO and NT-proBNP were positively associated with disease severity in patients with gestational hypertension, but HIF-1α was negatively correlated with disease severity (*P* < 0.05, Figure 2).

### Comparison of left ventricular function indexes in patients with different severity

**Figure 3:**
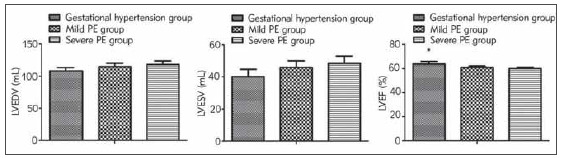
Comparison of left ventricular function indexes in patients with different severity. Comparison of LVEDV, LVESV and LVEF among the three groups, F=35.027, 35.301, 53.556, P < 0.05; vs. the mild PE and the severe PE, *P < 0.05.

The LVEDV and LVESV values of gestational hypertension patients were elevated with disease aggravation, but LVEF value was reduced (*P* < 0.05, Figure 3).

### Correlation analysis of serum TMAO, NT-proBNP, HIF-1α and left ventricular function indexes

**Figure 4:**
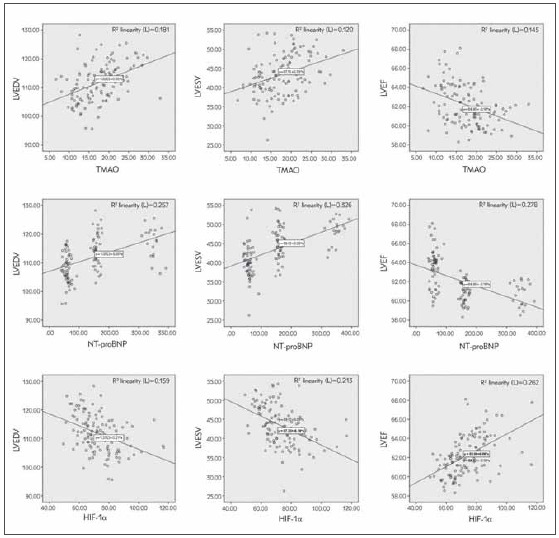
Correlation analysis between serum TMAO, NT-proBNP, HIF-1α and left heart function indexes

The serum TMAO, NT-proBNP, and HIF-1α were associated with LVEDV, LVESV, and LVEF values in patients with gestational hypertension (*P* < 0.05, Figure 4).

### Comparison of serum TMAO, NT-proBNP and HIF-1α between the good pregnancy outcome group and the poor pregnancy outcome

**Figure 5:**
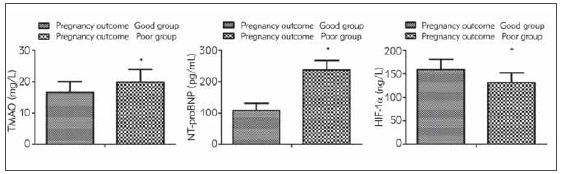
Comparison of serum TMAO, NT-probNP and HIF-1α between the better pregnancy outcome and the poor pregnancy outcome Vs. the better pregnancy outcome, *P < 0.05.

The serum TMAO and NT-proBNP were elevated but HIF-1α was reduced in patients with a poor pregnancy outcome compared with those with a good pregnancy outcome (*P* < 0.05, Figure 5).

### Analysis of the predictive value of serum TMAO, NT-proBNP and HIF-1α on pregnancy outcome

**Figure 6:**
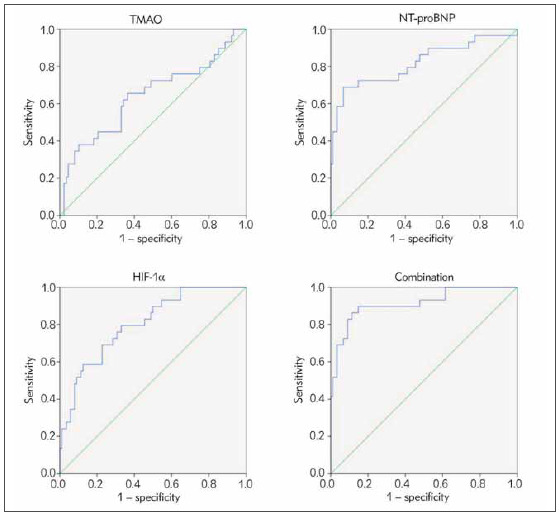
ROC curve analysis of serum TMAO, NT-proBNP and HIF-1α in predicting pregnancy outcome

The AUC of combined detection of serum TMAO, NT-proBNP and HIF-1α on prediction of pregnancy outcome was greater than that of detection of each indicator (*P* < 0.05, Table 2 and Figure 6).

**Table 2:** Predictive value analysis of serum TMAO, NT-probNP and HIF-1α on pregnancy outcome Note: Vs. the combination, * P < 0.06.

Indexes	Cut-off<br>point	AUC	SE	95%CI
TMAO, mg/L	17.85	0.640*	0.065	0.514∼0.767
NT-proBNP, pg/L	164.17	0.818*	0.054	0.713∼0.923
HIF-1a, ng/L	141.02	0.803*	0.045	0.715∼0.891
Combination		0.913	0.034	0.847∼0.980

## Discussion

Gestational hypertension mostly takes place after 20 weeks of pregnancy and 6 weeks after delivery. Its pathological changes are manifested as spasm of small blood vessels, which increases peripheral vascular resistance, resulting in decreased uteroplacental blood supply and placental function, which in turn causes fetal intrauterine growth retardation, which can lead to fetal death [8] [9]. At present, it is believed that the formation of hypertension is closely related to the formation of atherosclerosis, which can stimulate the thickness of the intima of the blood vessel, and the amount of oxygen entering the intima gradually reduces, which in turn leads to a reduction in serum HIF-1α [10]. Recent studies have clarified that the intestinal microbial metabolite TMAO can take part in the occurrence and development of atherosclerosis. TMAO can activate the NLRP3 inflammasome and motivate inflammation, which results in the occurrence of endothelial dysfunction [11] [12]. TMAO participates in oxidative stress, inflammatory response and the occurrence of atherosclerosis [13]. The prototype structure of NT-proBNP, brain natriuretic peptide, is the active precursor of polypeptides. Once the cardiomyocytes in the human heart are stimulated, they can be decomposed into NT-proBNP and amino acids under the action of activating enzymes. Plasma brain natriuretic peptide has no significant changes in different stages of gestational hypertension, which is difficult to apply to disease assessment, while NT-proBNP can be used to monitor cardiac function in patients due to its low plasma clearance rate. Studies suggest that it may be linked with disease progression in patients with gestational hypertension [13] [14]. This study discovered that serum TMAO, NT-proBNP were positively associated with the disease severity in patients with gestational hypertension, but HIF-1α was negatively associated. The reason is that the more severe the condition of patients with gestational hypertension, the heavier the heart load, the more likely to have cardiac insufficiency, resulting in the increase of serum NT-proBNP.

Hypertensive disorders of pregnancy can produce systemic small vasospasm, increase blood pressure, and increase cardiac load, resulting in a state of low output and high resistance, which in turn leads to a decrease in left ventricular diastolic function. Meanwhile, coronary artery spasm can result in myocardial ischemia interstitial edema in patients, leading to heart failure in severe cases [15] [16]. Relevant reports point out that the progression of the disease in patients with gestational hypertension is implicated in cardiac function [17]. This study discovered that LVEDV and LVESV values of patients with gestational hypertension were elevated with the severity of the disease, but the LVEF value was reduced, indicating that the cardiac function of patients with gestational hypertension is linked with the severity of hypertension, which is consistent with the results of a former study [18]. The results of this study clarified that serum TMAO, NT-proBNP, and HIF-1α in patients with gestational hypertension were linked with LVEDV, LVESV, and LVEF values, suggesting that serum TMAO, NT-proBNP, and HIF-1α were implicated in cardiac function in patients. This is because cardiac insufficiency leads to increased cardiac volume load and pressure, stretching of ventricular muscle fibers, which increases serum NT-proBNP. Increased cardiac load and blood flow resistance can increase myocardial oxygen consumption, cause tissue hypoxia, and lead to a decrease in HIF-1α. TMAO can activate inflammatory pathways, induce a variety of endothelial-related factors, and at the same time enhance the adhesion of macrophages, promote the occurrence of atherosclerosis, and ultimately affect hyperthe left ventricular function of patients [19] [20].

The main clinical manifestations of gestational hypertension are hypertension, proteinuria and edema. Patients are often accompanied by systemic multiple organ damage, and the disease will worsen with the progress of pregnancy. In severe cases, it will threaten the safety of the mother and the fetus [21] [22]. Some scholars have found that changes in cardiac function in patients with gestational hypertension are closely linked with pregnancy outcomes [23]. This study confirmed that the serum NT-proBNP of patients is related to the cardiac function of the patients, and it may be related to the pregnancy outcomes of the patients. Further research discovered that serum TMAO and NT-proBNP were promoted and HIF-1α was reduced in patients with a poor pregnancy outcome compared with those with a better pregnancy outcome. Moreover, the results of the research clarified that the AUC of combined detection of serum TMAO, NT-proBNP and HIF-1α on prediction of pregnancy outcome was greater than that of alone detection of each indicator, illustrating that combined detection had a predictive value for pregnancy outcome of patients with gestational hypertension.

All in all, serum TMAO, NT-proBNP and HIF-1α in patients with gestational hypertension are linked with disease severity, and have predictive and evaluative values for disease severity and pregnancy outcome.

## Dodatak

### Acknowledgments

Not applicable.

### Funding

Not applicable.

### Conflict of interest statement

All the authors declare that they have no conflict of interest in this work.
